# Video clips for patient comprehension of atrial fibrillation and deep vein thrombosis in emergency care. A randomised clinical trial

**DOI:** 10.1038/s41746-024-01107-7

**Published:** 2024-04-30

**Authors:** Santi Di Pietro, Ilaria Ferrari, Giuseppe Bulgari, Maria Lorenza Muiesan, Francesco Falaschi, Annalisa De Silvestri, Luigia Scudeller, Valeria Musella, Simone Saglio, Beatrice Re, Elena Mattiuzzo, Fabio Cherubini, Stefano Perlini, Improving Emergency Department Discharge Research Group, Clelia Alvich, Ernesto Anesi, Valentina Angeli, Bruno Barcella, Marco Bonzano, Giuseppe Bulgari, Maria Antonietta Bressan, Domenica Federica Briganti, Francesca Burlon, Valentina Carosio, Iride Ceresa, Fabio Cherubini, Giuseppe Crescenzi, Pietro Denti, Annalisa De Silvestri, Santi Di Pietro, Francesco Falaschi, Ilaria Ferrari, Roberta Guarnone, Barbara Guglielmana, Elisa Lainu, Elena Lago, Elena Maggi, Ilaria Malfasi, Ilaria Francesca Martino, Maria Mascolo, Elena Mattiuzzo, Giuseppe Mignosa, Maria Lorenza Muiesan, Valeria Musella, Ciro Paolillo, Giulia Perlini, Stefano Perlini, Pietro Pettenazza, Beatrice Re, Simone Saglio, Francesco Salinaro, Luigia Scudeller, Francesco Speciale, Ilaria Zunino

**Affiliations:** 1 Emergency Medicine Unit and Emergency Medicine Postgraduate Training Program, IRCCS Policlinico San Matteo Foundation, Department of Internal Medicine, University of Pavia, Pavia, Italy; 2 PhD Program in Experimental Medicine, University of Pavia, Pavia, Italy; 3 Emergency Department, Humanitas University Hospital, Rozzano, Italy; 4 Seconda Medicina Generale ASST Spedali Civili, Brescia, Italy; 5 Dipartimento di Scienza Cliniche e Sperimentali, Università di Brescia, Direttore 2° Medicina Generale ASST Spedali Civili, Brescia, Italy; 6 Assistance Publique Hôpitaux de Paris, Hôpital Antoine Béclère, GHU Paris-Saclay, Unité Polyvalente Aiguë de Court Séjour, Clamart, France; 7 Unit of Clinical Epidemiology and Biostatistics, Fondazione IRCCS Policlinico S. Matteo, Pavia, Italy; 8 Research and Innovation Unit, IRCCS Bologna University Hospital, Bologna, Italy; 9 Internal Medicine Residency Programme, University of Pavia, Pavia, Italy; 10 Respiratory Medicine Residency Programme, University of Milan, Milan, Italy; 11 Emergency Physician, Pavia Poison Centre, IRCCS Fondazione Salvatore Maugeri, Pavia, Italy; 12 Internal Medicine Residency Programme, University of Brescia, Brescia, Italy; 13 UOC Pronto Soccorso, Ospedale Civile Maggiore, DAI Emergenza e Terapie Intensive, Azienda Ospedaliera Universitaria Integrata (AOUI) di Verona, Verona, Italy

**Keywords:** Patient education, Randomized controlled trials

## Abstract

Integrating video clips in the discharge process may enhance patients’ understanding and awareness of their condition. To determine the effect of video clip-integrated discharge discussion on patient comprehension of atrial fibrillation (AF) and deep vein thrombosis (DVT), and their main complications (stroke and pulmonary embolism), we designed a multicentre, pragmatic, parallel groups, randomised clinical trial, that was conducted at two Emergency Units in Italy. A convenience sample of 144 adult patients (or their caregivers) discharged home with either AF or DVT were randomised to receive standard verbal instructions (control) or video clip-integrated doctor-patient discharge discussion. Participants were guided by the discharging physician through the clip. Mean score for primary outcome (knowledge of the diagnosis and its potential complication) (range 0–18) was 5.87 (95% CI, 5.02–6.72] in the control group and 8.28 (95% CI, 7.27–9.31) in the intervention group (mean difference, −2.41; 95% CI, −3.73 to −1.09; *p* < 0.001). Among secondary outcomes, mean score for knowledge of the prescribed therapy (range 0–6) was 2.98 (95% CI, 2.57–3.39) in the control group and 3.20 (95% CI, 2.73–3.67) in the study group (mean difference, −0.22; 95% CI, −0.84 to 0.39). Mean score for satisfaction (range 0–12) was 7.34 (95% CI, 6.45–8.23) in the control arm and 7.97 (95% CI, 7.15–8.78) in the intervention arm (mean difference, −0.625; 95% CI −1.82 to 0.57). Initiation rate of newly prescribed anticoagulants was 80% (36/45) in the control group and 90.2% (46/51) in the intervention group. Among 109 patients reached at a median follow up of 21 (IQR 16–28) months, 5.55% (3/54) in the control arm and 1.82% (1/55) in the intervention arm had developed stroke or pulmonary embolism. In this trial, video clip-integrated doctor-patient discharge discussion, improved participants comprehension of AF and DVT and their main complications. Physicians should consider integrating these inexpensive tools during the discharge process of patients with AF or DVT.

Trial Registration: ClinicalTrials.gov Identifier “NCT03734406”.

## Introduction

Giving patient discharge instructions is a key task of all health care professionals. To guarantee a safe discharge process, patients must have a good understanding of their medical condition, treatment, follow-up, and potential complications deriving from their condition or from newly prescribed treatments. This difficult task can be even more challenging when performed in some settings, such as the Emergency Department (ED), where the hectic environment, noise, frequent interruptions, and a high workload, can complicate patient education^1^. In Emergency Departments, discharge information is often delivered in a very short time – which in one study was found to be 76 seconds – therefore, it can be difficult for patients to understand and recall^1,2^. In addition to that, low health literacy, language barrier, altered cognitive function, or other patient-related factors, can further complicate communication and patient comprehension^3–6^. Several studies have demonstrated that discharge instructions are often incomplete^7^, and that patients frequently have deficits across several domains of comprehension^3,8,9^. Moreover, comprehension is a major predictor of compliance with discharge instructions^10^, which is in turn associated with patient outcomes^11,12^. Written instructions can improve recall, especially when information is simplified and combined with illustrations^13–15^. However, written instructions are not suitable for illiterate patients or caregivers and can be difficult to comprehend for those with low health literacy^6^. For these reasons, there is a growing interest in the adoption of video clips to deliver information and enhance communication during the process of discharge from the ED^16–23^. In a systematic review and meta-analysis conducted by Hoek et al., video discharge instructions showed the highest pooled recall (66.8%, 95% CI 57.9–75.7%) when compared to written (57.8%, 95% CI 44.2–71.2%) and verbal instructions (47%, 95% CI 32.2–61.7%), and the lowest variation in correct recall (*I*^2^ = 50.1%, 97.7%, 95.6% respectively), although these differences were not statistically significant^24^. Video clips and animations are also increasingly being used in a variety of clinical settings and across different disciplines to enhance communication between healthcare professionals and users. As an example, they can improve understanding of mechanical ventilation, its benefits, risks and alternatives among relatives of patients admitted to intensive care wards^25^. Animations can also improve understanding of procedures, as urgent angioplasty, or elective laparoscopic cholecystectomy, with the related benefits, risks, and alternatives to treatment^26,27^. By improving patient comprehension and awareness of a given medical treatment, and at the same time by helping healthcare professionals deliver evidence-based information, animations could help reconnect evidence-based medicine with shared decision making, to reach optimal patient care^28^. Most of the studies published on this topic utilised clips watched independently by patients or caregivers at discharge or immediately after discharge, whereas little is known about the effect of clips when these are integrated into doctor-patient (or caregiver) discharge conversation. We therefore conducted a multicentre randomised trial that investigated the effects of two clips shown by doctors to patients (or their caregivers) with atrial fibrillation (AF) and deep vein thrombosis (DVT) discharged home from two Emergency Units. We hypothesised that patients who receive video clip-integrated doctor-patient discharge discussion would show a higher comprehension of their condition and its complications.

## Results

### Participants and timeline

**Fig. 1:**
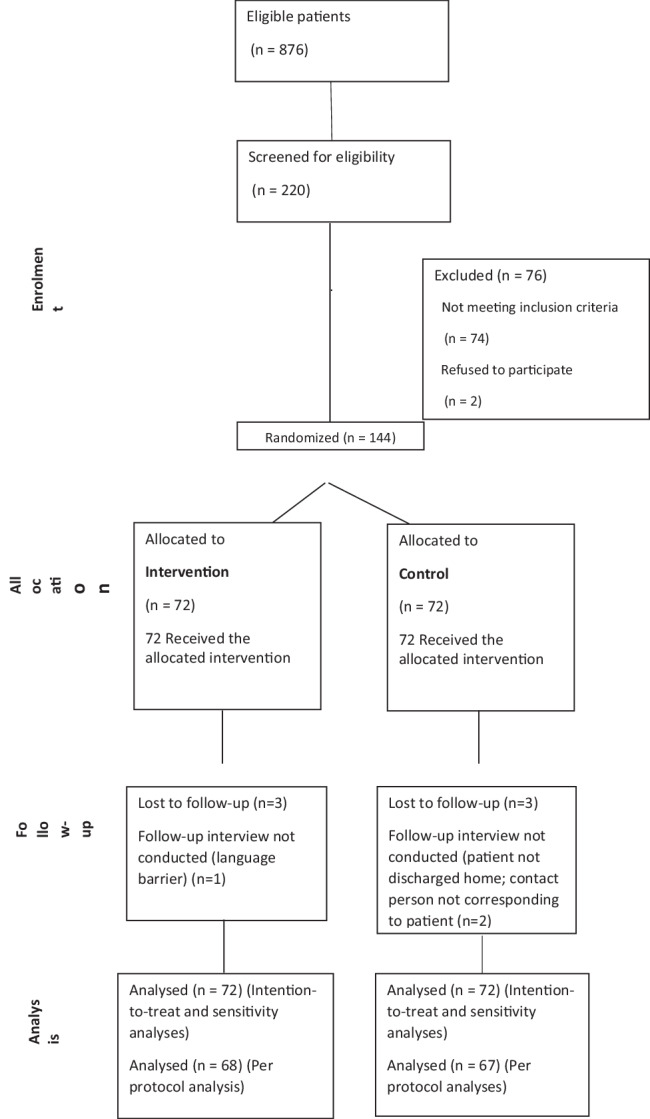
CONSORT flow diagram. The diagram provides a schematic illustration for the allocation and sequential flow of participants in our study, from enrollment to clinical trial analysis.

Between November 22, 2018, and December 27, 2021, a total of 876 eligible patients were seen at the recruiting centres. We screened for eligibility a convenience sample of 220 individuals and enroled 144 patients (or caregivers). Figure 1 illustrates the CONSORT flow diagram of the trial. Although the study was not formally suspended during the Covid-19 pandemic, no patients were enroled between February 2020 and October 2020, i.e., in the first and second waves of the pandemic. Of the 144 patients, 72 were randomly assigned to the intervention group and 72 to the standard group. Table 1 presents the characteristics of patients at baseline. The mean age (±SD) of the patients was 68.57 ± 13.96 years; 70 of the 144 patients were women (48.61%). Participants in the intervention group had a higher level of education (Table 1).

**Table 1:** Main characteristics of the patients at baseline

Characteristic	Standard group (*N* = 72)	Intervention group (*N* = 72)	Total (*N* = 144)
Age-yr	68.57 ± 13.96	68.53 ± 13.94	68.57 ± 13.96
Female sex-no. (%)	32 (44.44%)	38 (52.77)	70 (48.61)
Level of education-no. (%)^a^			
<5 years	21 (29.16)	21 (29.16)	42 (29.16)
5–8 years	20 (27.77)	8 (11.11)	28 (19.44)
8–13 years	24 (33.33)	37 (51.38)	61 (42.36)
>13 years	7 (9.72)	6 (8.33)	13 (9.02)
Medical condition-no. (%)			
AF^b^	68 (94.44)	65 (90.27)	133 (92.36)
DVT^c^	4 (5.55)	7 (9.72)	11 (7.63)

Plus-minus values are means ± SD.

^a^Level of education is reported as years of education.

^b^Atrial fibrillation.

^c^Deep vein thrombosis.

Six of the patients enroled were lost at follow-up (three for each arm). In three cases, the follow-up interview could not be conducted due to protocol violations (one in the intervention group, two in the control group). A total of 144 patients were included in the intention-to-treat analysis and in the sensitivity analyses, whereas 135 patients were included in the per protocol analysis.

There were 6 cases of protocol violation which are detailed in Table 2.

**Table 2:** Reasons for protocol violations and main characteristics of the patients

Cases of protocol violation	Study group	Age	Sex	Level of education	Reason for protocol violation
1	Intervention	94	F	8–13	Previous diagnosis of deep vein thrombosis
2	Intervention	45	M	<5	Language barrier – Not showing good understanding of Italian
3	Control	80	M	5–8	The person who received discharge instructions did not correspond to the one interviewed
4	Control	81	M	5–8	Previous diagnosis of deep vein thrombosis and pulmonary embolism
5	Control	80	F	5–8	Two previous episodes of atrial fibrillation and cardioembolic stroke
6	Control	72	M	<5	After discharge from the ED, he was transferred to a rehabilitation facility

### Effect of the intervention

Results for the primary and secondary outcomes are illustrated in Table 3.

**Table 3:** Outcomes of the trial

Outcome	Control Group (*N* = 72)		Intervention Group (*N* = 72)			
	No. of Patients	Mean (95% CI)	No. of Patients	Mean (95% CI)	Difference (95% CI)^a^	*P*-value
Knowledge of the diagnosis and its potential complication	72	5.87 (5.02–6.72)	72	8.29 (7.27–9.31)	−2.41 (−3.73 to −1.09)	<0.001
Knowledge of the prescribed therapy	72	2.98 (2.57–3.39)	72	3.20 (2.73–3.67)	−0.22 (−0.84–0.39)	0.47
Patient satisfaction	72	7.34 (6.45–8.23)	72	7.97 (7.15–8.78)	−0.62 (−1.82–0.57)	0.30

Possible scores ranged 0–18 for the main outcome; 0–6 for the knowledge of the prescribed therapy; 0–12 for patient satisfaction.

^a^Difference in the scores obtained by the two study groups, reported with 95% confidence interval.

These findings from the intention-to-treat analysis were confirmed in the per-protocol and sensitivity analyses, which can be accessed in Supplementary Data 5. Univariate and multivariate regression models showed that the primary outcome significantly correlates with several independent factors, including age (inverse correlation), level of education and having watched the video clip (Table 4). It was not feasible to analyse whether being diagnosed with DVT or AF had a different correlation with comprehension, due to the limited number of DVT cases.

**Table 4:** Factors associated with patient comprehension of their medical condition and its potential main complication

Predictor	Coefficient (95% CI)	*P*-value
Age	−0.08 (−0.13 to −0.04)	<0.001
Video clip-integrated discussion	2.48 (1.41–3.55)	<0.001
Level of education 8–13 years	1.58 (0.23–2.92)	0.02
Level of education >13 years	5.00 (2.80–7.20)	<0.001
Sex (Male)	−0.49	0.37

We analysed data of patients discharged home with a newly prescribed anticoagulant, to detect any difference in the initiation rate. Among all the interviewed patients, 96 were discharged with a new take-home anticoagulant. Anticoagulant initiation rate was 80% (36/45) in the control group and 90.2% (46/51) [x^2^ (1) = 1.99; *p* = 0.158] in the intervention group, a difference that did not reach statistical significance. We reached 109 of the recruited patients at a median follow up of 21 (IQR 16–28) months. Of these patients, 3.67% (4/109) had developed stroke or pulmonary embolism, respectively 5.55% (3/54) in the control arm and 1.82% (1/55) [Fisher’s exact test, *p* = 0.36] in the intervention arm, a difference that was not statistically significant.

## Discussion

In our randomised controlled trial, two short video clips integrated in doctor-patient discharge discussion, improved patient (or caregiver) comprehension of atrial fibrillation and deep vein thrombosis and their main complications. In line with previous studies^3,8,9^, patients and caregivers enroled in our trial showed on average a poor level of comprehension across all domains. Unsurprisingly, younger individuals and those with a higher level of education performed better than their counterparts, whereas integrating video clips into the discharge discussion was independently associated with better comprehension. Our findings – together with those from a growing number of trials^29^- strengthen the promising results of a recent meta-analysis, which suggests that adding video information to discharge instructions in EDs may improve comprehension and recall^21^. Unlike most of existing studies that utilised clips with audio content watched independently by patients or caregivers at discharge or immediately after discharge, we deliberately asked doctors to show the short clips to patients and comment on the video. One could criticise this choice as it can be time-consuming and make the discharge process less efficient; or it could be suggested that an audio content, developed in a rigorous way, may improve efficiency as compared to our approach. However, our study focus was on two high-risk medical conditions that can lead to serious complications and often require prescription of anticoagulants. Watching the clip may enrich doctor-patient conversation in several ways – with potential behavioural changes both on doctor and patient side. For example, it could help patients to formulate important questions about their health or about the proposed treatment; questions that we believe should be addressed and discussed during the discharge process, while the physician is still there to answer them, and not once the patient is at home. We do not suggest that this strategy of communication should be applied to all medical conditions, but it should be considered during selected high-risk discharge processes, when efficacy of communication and safety should be prioritised over time efficiency. Of course, a standardised audio content would decrease the variation of the verbal explanations provided to patients, but we preferred a pragmatic approach for this study. We asked physicians to illustrate the clips as they are typically involved in the discharge process at our institutions, in line with the pragmatic approach. Nevertheless, in other clinical settings, nursing staff or physician assistants oversee this task. Future studies should investigate whether the effect of clips can be replicated when these are delivered by other healthcare professionals. Although improved patient’s understanding and awareness of medical conditions is known to correlate with adherence to prescribed treatments, we failed to demonstrate higher initiation rate of anticoagulants in the intervention group. Nevertheless, it is definitely possible that our sample size was underpowered to detect this difference. Similarly, an even larger sample size would have been required to detect a difference, if any exists, in the rate of adverse events due to non-initiation or possibly non-adherence to anticoagulants. This study has several limitations. Firstly, the scoring tool used was not validated, as we could not find a tool that would allow the simultaneous measurement of different domains of comprehension across two different medical conditions. In addition to that, construct validation of our scoring system would require an amount of time and resources equivalent to that of the trial itself, pushing it beyond practical feasibility.

Secondly, despite the randomisation, individuals enroled in the intervention group had a higher level of education, a variable that may have affected the primary outcome by favouring higher comprehension scores in the intervention group. Thirdly, unblinding occurred in seven cases, as the person interviewed mentioned the video clip during the phone call, thus making the independent reviewers aware of the allocation arm. Fourthly, the number of protocol violations is unevenly distributed among the two arms, with more violations that occurred in the control group. This may have favoured the control group, as in most violation cases patients had already been diagnosed with AF or DVT, or had already developed stroke or pulmonary embolism, hence they were likely more skilled about their condition. Fifthly, doctors who were discharging patients may have inadvertently enhanced the quality of their verbal explanations -as compared to what happens in the normal clinical setting outside of a clinical trial- due to Hawthorne effect. Sixthly, in our trial patients were recruited both at Emergency Departments and Short-Stay Units, which may be considered as a study design limitation, as these settings can have a different tempo, noise level and stress level. Nevertheless, at our recruiting centres the Short-Stay Unit is adjacent to the resuscitation room and physicians in charge of this area also look after patients in other areas (including resuscitating severely ill patients or leading major trauma calls). Therefore, in our setting the stress level and time restraints of the discharge process are similar between these areas of the department. Although this study adds further knowledge on the effect of video clips when integrated in the discharge process, it does not explain how clips exert this effect and how they influence doctor-patient discussion. In particular, we do not know whether clips somehow affected the length of doctor-patient discussion; or the quality and completeness of doctor’s explanations – which in the case of AF is often imbalanced in the discussion of stroke vs bleeding risk and permeated by emotional language^30^; or the tendency of patients to pose questions and interact with physicians. All these questions should be addressed in future qualitative studies. In addition to that, future trials should inform about the external validity of our findings, by investigating the effect of video clips across different countries, languages, and healthcare systems. In conclusion, our study shows that when short clips are integrated into doctor-patient discussion, with an active involvement of physicians who illustrates the video content and use it as a visual support in adjunct to standard verbal instructions, patients (or caregivers) have a better understanding of AF and DVT and their main complications (stroke and pulmonary embolism). Therefore, we believe that physicians should consider integrating these inexpensive tools during the discharge process of patients with AF and DVT.

## Methods

### Trial design

This was a multicentre, pragmatic, parallel groups trial, with 1:1 randomisation, that aimed at assessing the effect of video clip-integrated doctor-patient discussion on patient comprehension of two medical conditions, namely atrial fibrillation and deep vein thrombosis.

The trial protocol and statistical analysis plan can be accessed in Supplementary Data 1.

This study followed the Consolidated Standards of Reporting Trials^31^ reporting guideline (CONSORT Checklist can be accessed in Supplementary Data 2).

This study was conducted in accordance with the ethical standards of the responsible committee on human experimentation (institutional and national) and with the Helsinki Declaration. The two relevant ethical committees, “Comitato Etico Pavia” and “Comitato Etico di Brescia”, approved this protocol, respectively on September 5, 2018, and on May 7, 2019. Informed consent was obtained from all human participants.

### Participants

We included a convenience sample of adult patients (≥18 years old) who were discharged home from one of two emergency departments with either atrial fibrillation or deep vein thrombosis and were considered capable of understanding the discharge information and explanations by the doctor in charge of this task. When patients were lacking capacity, or in all cases of patients judged by the treating doctor as not capable of safely receiving the discharge instructions, study participation was proposed to the caregiver with identical modalities.

Patients who required hospitalisation after being treated in the Emergency Units were not considered eligible. Similarly, we excluded patients with known history of deep vein thrombosis or two or more episodes of atrial fibrillation. Patients who had one previous episode of atrial fibrillation were considered eligible only if they had never been prescribed medications for its cure or prevention of recurrence (anticoagulants, antiarrhythmic drugs). A detailed list of inclusion and exclusion criteria is reported in the trial protocol in Supplementary Data 1.

The trial was conducted across two Emergency Units, namely the “*S.C. Pronto Soccorso Accettazione of the IRCCS Fondazione Policlinico San Matteo”* (Pavia, Italy) which includes the Emergency Department and its adjacent Short-Stay Unit, and the Emergency Unit *2° Medicina Generale, ASST Spedali Civili di Brescia* (Brescia, Italy).

### Interventions

During the discharge process subjects enroled in the study group were shown a clip related to their condition (DVT or AF), using it as a graphic support to the doctor’s verbal explanation of the diagnosed pathology and its potential main complication.

The clips last between 27 and 45 s and show the pathophysiological process underlying the two conditions under study (clips can be accessed in Supplementary video 1 and Supplementary video 2).

Clips were deliberately left without audio content, as it was specifically requested to the discharging doctor to describe in a structured manner what was shown on the screen, guiding patients during the vision of the clip. For a detailed description of the communication strategy please refer to the study protocol (Supplementary Data 1).

Although doctors followed a structured approach when explaining the video content, we did not aim to standardise the linguistic style of their explanations. As an example, they could use the term “thrombus” or “clot” or any other synonym they believed was suitable for the comprehension of the patient, even recurring to street slang or dialectal forms if deemed appropriate.

Patients enroled in the control group received discharge explanations without the aid of any video. Following a pragmatic approach, we asked doctors to express themselves in the way they are used to in their clinical practice, which is based on the doctor’s verbal and non-verbal communication skills. All participants (from both study arms) received standard written instructions.

Within 48 h from hospital discharge, all participants were administered a telephone interview that consisted of six questions, focusing on the different domains of comprehension and patient satisfaction (Supplementary Data 3). Audio recordings were then stored on a computer and subsequently re-examined by two independent reviewers, who assigned a score to each question contained in the interview.

An interpretation scheme was developed to aid the reviewers in the evaluation of patients’ answers, and to reduce the intra-operator and inter-operator variability (Supplementary Data 4).

### Outcomes

The main outcome was patient comprehension of the domains directly related to the video contents, i.e., knowledge of the diagnosis and its potential complication, corresponding to interview’s questions 1-3-4 (Supplementary Data 3). Secondary outcomes were knowledge of the prescribed therapy - assessed with question 2 of the interview - and patient satisfaction - corresponding to questions 5 and 6. Patients’ answers were scored on a 4-point Likert scale (ranging from zero to three) from low to high knowledge or low to high satisfaction. Scores from the two independent reviewers were summed to obtain an overall score. Therefore, the score ranged: 0–18 for the main outcome (questions 1-3-4), 0–6 for the knowledge of the prescribed therapy (question 2), 0–12 for patient satisfaction (questions 5–6).

In the second version of the protocol two safety outcomes were added. We were interested in measuring anticoagulation initiation rate among patients discharged home with newly prescribed anticoagulants. Data for this outcome were obtained from a further review of the audio recording performed by one of the two reviewers. In addition to that, we measured the incidence of the main complication (stroke/pulmonary embolism) at a median follow up median follow up of 21 (IQR 16–28) months, assessed with a second telephone contact with recruited participants. During this second telephone contact we did not assess adherence to anticoagulants. The addition of these safety outcomes was made during the recruitment phase. This was felt necessary due to relatively frequent patient-reported non-initiation of anticoagulants observed during telephone interviews.

Although the two clips per se do not contain any visual information on medications (effect, side effects, interactions etc.) or any explicit message supporting adherence to anticoagulants, we hypothesised that there could be indirect effects of integrating the clips into the discharge conversation, which we explored in the secondary outcomes.

### Sample size

We hypothesised a median score for the primary outcome to be 10 points in the control group, whereas we expected a 20% higher score in the intervention group as suggested by the existing literature with standard deviation 4 and non-normal distribution. Group sample sizes of 64 and 64 would achieve 80% power to detect this difference with alpha error 5% using a two-sided Mann-Whitney test assuming that the actual distribution is uniform.

Since we expected the number of patients lost to follow-up to be 10%, we planned to enrol 72 patients in each group (144 patients in total). We did not plan interim analyses for this trial.

### Randomisation

Opaque envelopes were used in the emergency units by the discharging doctors to assign recruited patients to control or study groups.

The statistician disposed the randomised sequence through the generation of pseudo-random numbers, divided in blocks of variable size and stratified for the participating institutions.

The statistician also prepared the opaque envelopes containing the progressive sequence of enrolment and the allocation of the patient.

### Blinding

It was not feasible to blind patients or the discharging doctor. However, the doctor responsible for the discharge process asked patients in the intervention group to not mention during the telephone interview the video clip they had watched. In addition to that, investigators conducting the interviews never mentioned the video clips. By doing that we obtained blinding of the two independent reviewers who assessed the audio recordings of the interviews.

### Statistical methods

We performed the main analysis with an intention-to-treat perspective, considering patients lost to follow-up as having score zero.

A secondary analysis was performed “on treatment” (per protocol), i.e., on patients who have been reached at the follow-up call within 48 h from the discharge.

In addition to that, we also conducted a sensitivity analysis, considering patients lost to follow-up as “average” knowledge (inputting the missing score with mean score across both groups).

Descriptive statistics was used for all variables assessed in the study population. Mean and standard deviation were used for normally distributed variables, mean and interquartile range for skewed distributions, proportions for categorical variables. Whenever relevant, 95% confidence intervals (95% CI) were calculated.

Groups were compared by means of parametric or nonparametric tests for quantitative variables (according to distribution; normality will be tested by means of the Shapiro–Wilk test) and Pearson’s χ2 test (Fisher exact test where appropriate) for categorical variables. In all cases, two-tailed tests were applied. *P*-value < 0.05 was considered significant. Bonferroni correction was used whenever relevant.

Factors potentially associated to knowledge and to patient satisfaction, including age, gender, comorbidities, years of education were tested by means of univariate and multivariate quantile (median) regression models.

All statistical analyses were conducted using Stata computer software version 15 (Stata Corporation, 4905 Lakeway Drive, College Station, Texas 77845, USA).

### Supplementary information

Supplementary information

Supplementary Video 1

Supplementary Video 2

## Data Availability

The data that support the findings of this study are available from the corresponding author upon reasonable request.
