# Supplementary material for: Video clips for patient comprehension of atrial fibrillation and deep vein thrombosis in emergency care. A randomised clinical trial
Source: NPJ Digit Med. 2024 Apr 30;7:107. doi: 10.1038/s41746-024-01107-7 (PMC11061292; doi:10.1038/s41746-024-01107-7)
Supplement: Supplementary file 1 — Supplementary information [file 41746_2024_1107_MOESM1_ESM.pdf]

Supplementary Data 1

Protocol version 2.0 (15 September 2021)

Can video clips improve patient comprehension at the Emergency Department? Protocol of a Multicenter Randomized Controlled Trial.

Name and affiliations of Sub-investigators

The definitive list of sub-investigators will be available when patients' enrolment will be completed. In fact, as patient recruitment proceeds more sub-investigators are supporting the enrolment process.

Roles and Responsibilities

We appointed a 'Principal Investigator' (PI), for each centre involved in the study, and a Clinical Research Coordinator (CRC); they will coordinate the enrolment process at their centre and guarantee the safekeeping of patient's files (the informed consents obtained and patient's discharge letters).
The PI of the study in Pavia is IF, whereas the CRC is SDP; the PI of the study in Brescia is MLM whereas GB is the CRC.
'Sub-investigators' (SI) are consultant and resident physicians working in the Emergency Department (ED), who have accepted to participate in the study. SIs are responsible for the enrolment and the randomization of patients in the emergency department with the modalities described in the protocol. All patients enrolled in the study group, will be shown by SIs the interventional video related to their medical condition, using SI's personal device (tablet / smartphone / notebook) or computers in use within the Department. Moreover, SIs oversee collecting and safekeeping patient's personal data, which are kept in a safe place within their institution agreed with the local PI.

The SIs attend a preliminary training session conducted by PI and CRC in the participating centres. Several training sessions have been organized at each centre, to allow all consultants and residents to take part during their time off from clinical duties.

The preliminary training is intended to instruct SIs in all aspects of the protocol, including study proposals to the patients, appropriate use of the videos and sensitive data storage.

The efficacy of the interventional videos will be assessed through telephone interviews that are conducted within 48 hours from patient's discharge from the ED. All telephone interviews are conducted by the PI or the CRC in Pavia.

Before the beginning of the study, the two researchers have defined and standardized the communication strategy to adopt during interviews, paying particular attention not to influence the opinion of the respondents. The exact content of the interview is not shared with SIs, to avoid them involuntarily preparing patients for the questions they will be asked during the interview.

The two interviewers store the recordings of telephone calls onto an institutional computer, where they will be available for the review process.

Each telephone interview will be reviewed by two independent physicians (FF and FS). Both will process all interviews independently, assigning scores to the patient's answers based on their level of comprehension.

Reviewers will report these data on a paper form and then transfer them onto the collection software. IF and SDP have instructed the reviewers, as well as all other researchers involved in the study.

Introduction

Background and rationale

Despite the remarkable technological progress of modern medicine, communication between patients and doctors is still an issue today. Trying to guarantee a concise, complete and transparent communication of information, possibly appropriate to the educational level of the patient, remains nowadays as in the past, a complex challenge for the clinician.

In the context of Emergency Departments (EDs), this challenge is made even more difficult due to the presence of various factors: overcrowding, simultaneous management of multiple patients, lack of privacy, noise, interruptions, and time constraints [[1],[2]].

Additional patient-specific elements can further complicate patient-provider communication: cultural differences, language barriers, differences in educational levels and demographics, etc. [[3],[4]].

Furthermore, both medical school and higher specialty training in Emergency Medicine currently dedicate little time to the development of communication skills: however, these techniques can be learned by doctors along with technical skills, which are usually prioritized in the educational curricula of trainees [5] [1,6].

A task force of the 'Society for Academic Emergency Medicine' has identified five essential elements in patient-provider communication in the Emergency Department: establishing rapport, providing information, providing comfort, creating a collaborative relationship and gather information [7].

Current literature on this subject shows that doctors' explanations at discharge are often deficient in one or more of the aforementioned areas [8,9]. In one study, the average duration of doctors' explanation of discharge instruction was 76 seconds, due to overcrowding and time constraints [8].

Other studies have shown that ED-patients' comprehension often shows deficiencies across several domains, such as diagnosis and its causes, ED care, post-ED care, return instructions, etc.[10-11].

Comprehension is the major predictor of compliance with discharge instructions [12], and compliance with discharge instructions has been associated with better patient outcomes [13,14].

All this evidence highlights the need for effective interventions aimed at improving communication in emergency departments.

A 2012 review on the topic summarizes some of the various solutions described in the current literature such as the standardization of discharge instructions in a diagnose-specific manner and the simplification of their language content [15].

Graphic illustrations or "comics" have showed benefits in the comprehension of patients in a variety of settings, including the discharge from ED [16,17]. Similarly, the use of video clips has been investigated in few studies, both in adult and pediatric EDs, showing promising results [18,19,20,21].

Objectives

The aim of this study is to assess the utility of two interventional videos, shown to patients discharged from the ED with a diagnosis of atrial fibrillation or deep vein thrombosis. We have focused our attention on these two medical conditions because they share some important features: they are frequently seen in EDs; their main complication (stroke and pulmonary embolism) can cause significant morbidity and mortality; both conditions and their related complications are probably not

very intuitive to comprehend for the general population. In fact, the understanding of these conditions implies the knowledge of a number of pathological processes, with which most people are unfamiliar.

The primary hypothesis is that patients enrolled in the study group will show a higher degree of comprehension of their medical condition and its related complications, as compared to the control group. Secondly, we will also analyze any possible effect of the videos on the knowledge of the therapy prescribed, return instructions and patients' satisfaction with the quality of communication in the ED.

Eventually, we will examine the association of some factors with the outcomes (age, sex, level of education, atrial fibrillation vs. deep vein thrombosis group).

Trial design

We designed a multicentric, pragmatic, parallel groups trial, with 1:1 randomization, that involves patients discharged from two Emergency Units with a diagnosis of atrial fibrillation (AF) or deep vein thrombosis (DVT).

The study aims to demonstrate the superiority of the intervention, assessed with a telephone interview that is conducted within 48 hours from patient's discharge.

This protocol was developed following the SPIRIT checklist.

Methods: Participants, interventions, and outcomes

Study setting

The trial takes place two Emergency Units, namely the "*S.C. Pronto Soccorso Accettazione of the IRCCS Fondazione Policlinico San Matteo"* (Pavia, Italy) which includes the Emergency Department and its adjacent Short-Stay Unit, and the Emergency Unit *2° Medicina Generale, ASST Spedali Civili di Brescia* (Brescia, Italy).

Both institutions are tertiary referral hospitals, serving as "Hub" centres for STEMI, Stroke and Trauma patients.

Eligibility criteria

Inclusion criteria are the following:

- Age >18
- Being diagnosed with one of the two medical conditions under study: patients with first-time diagnosis of deep vein thrombosis; patients with first-time diagnosis of atrial fibrillation; patients with second episode in life of atrial fibrillation who have never been prescribed take-home medications for its cure or prevention of recurrence (anticoagulants, antiarrhythmic drugs including beta-blockers)
- Being discharged home directly from the Emergency Department/Emergency Unit
- Capacity of the patient to understand the explanations and instructions provided by the doctor (this includes excellent command of the Italian language)
- Having provided a valid phone number
- Having signed the informed consent

In case of patients lacking capacity, and in all cases of patients judged by the recruiting doctor as incapable to safely receive the discharge instructions, the caregiver will be enrolled to the study with identical modalities.
In the context of a pragmatic trial, we did not set exclusion criteria in terms of education level and comorbidities of the enrolled patients.

Exclusion criteria:

- Previous diagnosis of deep vein thrombosis
- Two or more previous episodes of atrial fibrillation; or one previous episode of atrial fibrillation for which the patient has been prescribed one of the following take-home medications: anticoagulants, antiarrhythmic drugs including beta-blockers.
- Being hospitalized

Interventions

Study group

All subjects enrolled in the study group are shown during the discharge process the video related to the patient's condition (DVT vs AF), using it as a graphic support to the doctor's verbal explanation of the diagnosed pathology and its potential main complication.

For the purposes of the study we have selected two video clips, available on various internet sites and not covered by any copyright restrictions; these have been modified and shortened to make them suitable to use in our study setting.

The clips last between 27 and 45 seconds and show the pathophysiological process underlying the two diseases under study, namely deep vein thrombosis and atrial fibrillation.

The SIs show the videos to patients on the institutional computer or, alternatively, on their personal smartphone / tablet (in any case in resolutions from 480 pixels to 720 pixels). The content of these video clips is adequate for adults with all levels of education. In addition to that, videos are purposely without audio content, as it is specifically requested that SIs provide explanations and comments according to what is shown on the screen, guiding patients during the vision of the clip. In particular, SIs are asked to structure the explanation as follows: a) describe the relevant anatomical structures and the physiological processes in normal conditions (lower limb veins and venous circulation/heart chambers and normal blood flow between atria and ventricles); b) describe the pathological process that determines formation of a blood clot in the respective anatomical sites (stagnation of blood in proximity of venous valves with formation of a thrombus/ fibrillation of the right atrium, stagnation of blood and formation of a thrombus in the atrium); c) describe the dislocation of the thrombus to reach the target anatomical site (lung/brain); d) describe the complications derived from clot's dislocation to the target anatomical site (pulmonary embolism/stroke).

Although doctors follow the steps described above, which are based on the structure of the videos, we did not aim to standardize the linguistic style of their explanations. As an example, they could use the term "thrombus" or "clot" or any other synonym they believe is suitable for the comprehension of the patient, even recurring to street slang or dialectal forms if deemed appropriate.

Doctors can pause and rewind the clip at their discretion or if the patient requests to do so.

All patients will receive standard written discharge instructions.

Control group

Patients of the control group receive discharge explanations without the aid of any video or graphic support. Following a pragmatic approach, we have asked doctors to express themselves in the way

they are used to in their clinical practice, which is based on the doctor's verbal and non-verbal communication skills.
All patients will receive standard written discharge instructions.

Participating medical personnel

The SIs, i.e., the doctors responsible for the recruitment of patients, are selected on a voluntary basis among the consultants and residents working in the emergency departments during the enrolment stage. All consultants and residents at each institution are invited to participate to the study, without any restrictions in terms of years of experience within the same role.
SIs are asked to participate to the preliminary training and to conduct the research activities always in accordance with the study protocol.
One of two doctors in charge of reviewing telephone interviews (FF) works in an Internal Medicine ward therefore does not interact with patients recruited. The other reviewer (FS) who works at the Emergency Department in Pavia is not allowed to enroll patients.

Outcomes

The main outcome will be patients' comprehension of the domains directly related to the video contents, i.e., knowledge of the *diagnosis* and its potential *complication*, corresponding to interview's questions 1-3-4 (see the attached file *Telephone Interview*).
Secondary outcomes will be the knowledge of the prescribed therapy and patients' satisfaction (see the attached file *Telephone Interview*).
The outcomes will be measured through a telephone interview, consisting of a set of questions specifically designed for the purposes of this study (see the attached file "*Telephone interview*"). The interview is made of six questions, focusing on the different domains of comprehension.
The telephone interview is conducted by one of the two researchers responsible for this task. Audio files are then stored on a computer and subsequently reexamined by both reviewers, who will assign a score to each question contained in the interview (see the attached file "*Telephone interview*").

We developed a scheme to aid the reviewers in the interpretation of patients' answers, to reduce reviewers' inter-operator variability (see the attached file *Interpretation of patients' answers*).
Patients' answers will receive a score on a 4-point Likert scale (ranging from zero to three) from low to high knowledge.
Scores from the two independent reviewers will be summed to obtain an overall score. The score for the main outcomes will therefore range: 0-18 for the main outcome (questions 1-3-4), 0-6 for the knowledge of the prescribed therapy (question 2), 0-12 for patient's satisfaction (questions 5-6).

To the best of our knowledge, no currently available validated questionnaires would serve for the purpose of our study, i.e., for the simultaneous measurement of different domains of comprehension across two different medical conditions.
Hence, we have developed the ad-hoc interview and its related interpretation scheme using face and content validity. Construct validation would require an amount of time and resources equivalent to that of the trial itself, pushing it beyond practical feasibility.

In this second version of the protocol, we added two safety outcomes. The first is patient adherence to anticoagulation prescribed at hospital discharge. The second outcome is incidence of the main complication (stroke/pulmonary embolism) at a median follow up. This amendment is felt necessary due to relatively frequent patient-reported non-adherence to anticoagulants observed during telephone interviews.
Data for adherence rate to anticoagulants will be obtained from an additional review of the audio recording of the interview. This will be performed by one of the two reviewers.
Incidence of complications at median follow up will be assessed via a second telephone contact with recruited participants (patients or caregiver), using the same contact information provided at the time of recruitment. We will make a maximum of five attempts of contact, after which participants will be considered lost at follow up.

Participants timeline

Patient enrolment will take place in the emergency departments without any time restriction, including night shifts and festivities. Nevertheless, it is possible that the enrolment of eligible patients does not occur in a continuous fashion, as not all the doctors serving in the department participate in the study.

Prior to the discharge of eligible patients, the SIs will invite patients (or caregivers) to participate to the study, explaining its purpose and the methods used. Patients (or caregivers) willing to participate will sign the consent, provide a valid telephone number and are assigned to the study or control group. After patient's discharge, the SIs will transmit the telephone number to the PIs in charge of conducting interviews.

Telephone interviews will be carried out within 48 hours from discharge, using an institutional telephone station located in the emergency department of the IRCCS Fondazione Policlinico San Matteo Hospital.

The telephone station is connected to the institutional computer situated in the same room, which is provided with a free downloadable software for phone call management. All recordings will be stored on the same computer and each file is named with a progressive number code.

The equipment used to carry out phone interviews are situated in the same room where all files related to the study will be stored (consents, discharge letters, opaque envelopes for randomization).

Interviewers will try to contact patients (or caregivers) telephonically with a maximum of five attempts, after which the patients will be considered lost to follow-up.

During a patient's permanence within the emergency department, the SIs do not anticipate to the patient the content of the telephone interview, in order not to influence their answers.

Sample size

We hypothesize a median score for the primary outcome to be 10 points in the control group, whereas we expect a 20% higher score in the intervention group as suggested by current literature, with standard deviation 4 and non-normal distribution. Group sample sizes of 64 and 64 achieve 80% power to detect this difference with alpha error 5% using a two-sided Mann-Whitney test assuming that the actual distribution is uniform. (Note: this number represents the total number of patients with either DVT or AF, within each study group, since we do not expect differences in the effect by diagnosis).

Since we expect the number of patients lost to follow-up to be 10%, we plan to enroll 72 patients in each group (144 patients in total).

Methods: assignment of interventions

Allocation – Sequence generation

The statistician (LS) has disposed the randomized sequence through the generation of pseudo-random numbers, divided in blocks of variable size and stratified for the participating institutions.
The statistician has also prepared the opaque envelopes containing the progressive sequence of enrolment and the allocation of the patient.
Opaque envelopes are used in the emergency department by the SIs to assign patients to control or study group.

Blinding

Blinding is not applicable to the SIs who have to enroll and randomize patients.
SIs ask patients enrolled in the intervention group not to mention the video clip when they are contacted for the telephone interview. Similarly, investigators conducting the interview never mention the content of the video. These two strategies allow for blinding of the two independent reviewers.

Methods: data collection, management, and analysis

Data collection instruments

Source documents

Study data are collected on source documents. The PI is responsible for assuring that collected data are complete and accurate. Source documentation (the point of initial recording of a piece of data) supports data collected on the eCRF. Source documents include all recordings of observations or notations of clinical activities and all reports and records necessary for the evaluation and reconstruction of the clinical study. Completed eCRF data are entered into the database within a week of the patient visit being completed.

Data collection and study report form monitoring

All data obtained from this study are entered into a local regulation compliant Data Management System [for reference ex: 21 CFR Part 11 (USA)]. This is provided by the Service of Biometry & Clinical Trial Center (CTC) of the I.R.C.C.S. Policlinico San Matteo Foundation (Pavia, Italy). Data are recorded with an Electronic Data Capture (EDC) system using eCRFs. Specifically, the EDC is

based on the RedCap platform. REDCap is a novel workflow methodology and software tool that expedites the electronic collection of research data from a single site or multi-site clinical research study. The software supports a secure web-based application for developing fully functional case report forms (CRFs) and surveys. In particular, through RedCap we implement: (a) Full user authentication (log-on/password) to restrict users to study functions; (b) Real-time data validation, integrity checks for insuring data quality; (c) De-identification options to be applied to data exports to remove fields that contain notes and other information that could identify patient; (d) Centralized, secure storage of research data with back-ups; (e) The study database will be resident on a server in a secure location within the I.R.C.C.S. Policlinico San Matteo Foundation, Pavia, Italy.
The PIs will ensure the accuracy and completeness of the data reported to the DMC. All data entry, modification or deletion is recorded automatically in an electronic audit trail. The PIs will retain all copies of the eCRF in the relevant sections of their Investigator Site File with any required anonymised background information from the medical records as required.

Data management

All the consents of the enrolled patients and a copy of the discharge letters are stored in a safe site in each participating institution, where they will be available to the PIs for data monitoring and analysis. The reviewers will conduct their task in the same site where data are stored.
Once assessed each telephone interview, they fill out the paper sheet for score assignment and then transfer all data to the eCRF.

Statistical methods

A full data management plan and a full statistical analysis plan (SAP) has been revised and approved by the scientific board prior to study start. The SAP does not include details of data on rate of adverse events and decision rules for arm(s) or study termination, as this is not appropriate to this study. After SAP signature by the scientific board (i.e. prior to any statistical analysis), the study database will be locked. Any changes to the protocol-specified or SAP-specified planned analyses that are made after the database lock will be described in the clinical study report.

Analysis strategy

The main analysis will be performed with an ITT perspective, considering patients lost to follow-up as having score zero.

A secondary analysis will be performed "on treatment", i.e. on patients who have been reached at the follow-up call.

A sensitivity analysis will also be performed, considering patients lost to follow-up as "average" knowledge (inputting the missing score with mean score across both groups).

Statistical analyses

Descriptive statistics will be obtained for all variables assessed in the study population. Mean and standard deviation will be used for normally distributed variables, mean and interquartile range for skewed distributions, proportions for categorical variables. Whenever relevant, 95% confidence intervals (95% CI) will be calculated.

Groups will be compared by means of parametric or nonparametric tests for quantitative variables (according to distribution; normality will be tested by means of the Shapiro–Wilk test) and Pearson's $\chi 2$ test (Fisher exact test where appropriate) for categorical variables. In all cases, two-tailed tests will be applied. P-value <0.05 will be considered significant. Bonferroni correction will be used whenever relevant.

Factors potentially associated to knowledge and to patient's satisfaction, including age, gender, comorbidities, years of education will be tested my means of univariate and multivariate quantile (median) regression models.

Stata computer software version 15 or higher (Stata Corporation, 4905 Lakeway Drive, College Station, Texas 77845, USA) will be used for statistical analysis.

Ethics and dissemination

The two relevant ethical committees, "*Comitato Etico Pavia*" and "*Comitato Etico di Brescia*", have approved this protocol, respectively on September 5, 2018 and on May 7, 2019.

All patients or caregivers sign the informed consent prior to study enrolment and randomization.

Once completed the list of SIs, the PIs will communicate their names and affiliations to the Ethical Committees. It may happen during the conduction of telephone interviews, that patients demonstrate a potentially hazardous comprehension deficit of the prescribed therapies, e.g. a patient who ignores or refuses the prescription of parenteral or oral anticoagulants. In such cases, the interviewing PIs will promptly address the patients to his/her GP or send him/her back to the emergency department.

Dissemination policy

Patient's data will not be shared with third parties and will always be used respecting their privacy.
At the end of the study, PI's will produce a scientific paper for submission to peer-reviewed journals in the field of general medicine or emergency medicine.
In case of publication, PIs will participate as authors of the paper, whereas all participating SIs will result as contributors.
Manuscript preparation will follow the CONSORT guideline: http://www.equator-network.org/resource-centre/library-of-health-research-reporting/reporting-guidelines/. The authors of the publications will be decided on the basis of indications contained in the Uniform Requirements for Manuscripts (http://www.icmje.org/urm_full.pdf).

Funding

This study is not sponsored.
The conduction of the study does not add additional costs for the participating institutions.
The interventional videos are freely available on several websites and are not covered by copyright or royalties. Phone calls are made from a telephone within the IRCCS Ospedale San Matteo at negligible costs.
The software used for phone calls management is also freely downloadable from the internet.
Since the intervention under study is an educational video and not a drug, we will not stipulate any insurance, as the intervention does not constitute a potential harm for patients.

Bibliography

[1] Rosenzweig S. Emergency Rapport . J Emerg Med. 1993; 11:775-8.

[2] DeShazo RD. Two-way Medicine: Strategies for Improving Doctor-Patient Relationships. South Med J. 1993; 86(1):27-30.

[3] DeVoe JE, Wallace LS, Fryer GE Jr. Measuring patients' perceptions of communication with healthcare providers: do differences in demographic and socioeconomic characteristics matter? Health Expect. 2009;12:70-80.

[4] Anita Vashi, MD, MPH, Karin V. Rhodes, MD, MS. "Sign Right Here and You're Good to Go": A Content Analysis of Audiotaped Emergency Department Discharge Instructions. Ann Emerg Med. 2011;57:315-322.

[5] Epstein RM, Campbell TL, Cohen-Cole SA, et al. Perspectives on Patient-Doctor Communication. J Fam Pract. 1993;37(4):377-88.

[6] Novack DH, Volk G, Drossman DA, et al. Medical Interviewing and Interpersonal Skills Teaching in US Medical Schools. JAMA. 1993; 269(16):2101-5.

[7] Torten VL, Knopp R, Helpem K, et al. Physician-patient communication in the emergency department, part 2: communication strategies for specific situations. SAEM Task Force on Physician-Patient Communication. Acad Emerg Med. 1996;3:1146-1153

[8] Rhodes KV, Vieth T, He T, et al. Resuscitating the physician-patient relationship: emergency department communication in an academic medical center. Ann Emerg Med. 2004;44:262-267.

[9] Anita Vashi, MD, MPH, Karin V. Rhodes, MD, MS."Sign Right Here and You're Good to Go": A Content Analysis of Audiotaped Emergency Departement Discharge Instructions. Ann Emerg Med. 2011;57:315-322.

[10] John A. Crane, MD. Patient Comprehension of Doctor-Patient Communication on Discharge from the Emergency Department. The Journal of Emergency Medicine, Vol 15, No 1, pp 1-7, 1997

[11] Kirsten G. Engel, Michele Helsler, Dylan M. Smith, Claire H. Robinson, Jane H. Forman, Peter A. Ubel. Patient Comprehension of Emergency Departement Care and Instructions: Are Patients Aware of When They Do Not Understand? Ann Emerg Med. 2009;53:454-461

[12] Clarke C, Friedman SM, Shi K, Arenovich T, Monzon J et al. (2005) Emergency department discharge instructions comprehension and compliance study. CJEM 7: 5-11.

[13] Jack BW, Chetty VK, Anthony D, Greenwald JL, Sanchez GM et al. (2009) A reengineered hospital discharge program to decrease rehospitalization: a randomized trial. Ann Intern Med 150: 178-187. doi:10.7326/0003-4819-150-3-200902030-00007.

[14] VanSuch M, Naessens JM, Stroebel RJ, Huddleston JM, Williams AR (2006) Effect of discharge instructions on readmission of hospitalised patients with heart failure: do all of the Joint Commission on Accreditation of Healthcare Organizations heart failure core measures reflect better care? Qual Saf Health Care 15: 414-417.

[15] Margaret E. Samuels-Kalow, Anne M. Stack, Stephen C. Porter. Effective Discharge Communication in the Emergency Departement. Ann Emerg Med. 2012;60:152-159.

[16] Brand A, Gao L, Hamann A, Crayen C, Brand H, Squier SM, Stangl K, Kendel F, Stangl V. Medical Graphic Narratives to Improve Patient Comprehension and Periprocedural Anxiety Before Coronary Angiography and Percutaneous Coronary Intervention: A Randomized Trial. Ann Intern Med. 2019 Apr 16;170(8):579-581. doi: 10.7326/M18-2976. Epub 2019 Apr 9. PMID: 30959523.

[17] Paul E Austin, Robert Matlack II, Kathleen A Dunn, Charles Kesler, Charles K Brown. Discharge Instructions: Do Illustrations Help Our Patients Understand Them? Ann Emerg Med March 1995;25:317-320.

[18] Atzema CL, Austin PC, Wu L, et al. Speak fast, use jargon, and don't repeat yourself: a randomized trial assessing the effectiveness of online videos to supplement emergency department discharge instructions. PLoS One. 2013;8(11):e77057. Published 2013 Nov 11. doi:10.1371/journal.pone.0077057

[19] Choi S, Ahn J, Lee D, Jung Y. The Effectiveness of Mobile Discharge Instruction Videos (MDIVs) in communicating discharge instructions to patients with lacerations or sprains. South Med J. 2009 Mar;102(3):239-47. doi: 10.1097/SMJ.0b013e318197f319. PMID: 19204614.

[20] Baker MD, Monroe KW, King WD, Sorrentino A, Glaeser PW. Effectiveness of fever education in a pediatric emergency department. Pediatr Emerg Care. 2009 Sep;25(9):565-8. doi: 10.1097/PEC.0b013e3181b4f64e. PMID: 19755888.

[21] Ismail S, McIntosh M, Kalynych C, Joseph M, Wylie T, Butterfield R, Smotherman C, Kraemer DF, Osian SR. Impact of Video Discharge Instructions for Pediatric Fever and Closed Head Injury from the Emergency Department. J Emerg Med. 2016 Mar;50(3):e177-83. doi: 10.1016/j.jemermed.2015.10.006. Epub 2016 Jan 21. PMID: 26806318.

Supplementary Data 2

CONSORT 2010 checklist of information to include when reporting a randomised trial*

Section/Topic	Item No	Checklist item	Reported on page No
**Title and abstract**			
	1a	Identification as a randomised trial in the title	1
	1b	Structured summary of trial design, methods, results, and conclusions (for specific guidance see CONSORT for abstracts)	2-4
**Introduction**			
Background and objectives	2a	Scientific background and explanation of rationale	4
	2b	Specific objectives or hypotheses	4
**Methods**			
Trial design	3a	Description of trial design (such as parallel, factorial) including allocation ratio	5
	3b	Important changes to methods after trial commencement (such as eligibility criteria), with reasons	NA
Participants	4a	Eligibility criteria for participants	5
	4b	Settings and locations where the data were collected	5
Interventions	5	The interventions for each group with sufficient details to allow replication, including how and when they were actually administered	5-6
Outcomes	6a	Completely defined pre-specified primary and secondary outcome measures, including how and when they were assessed	6-7
	6b	Any changes to trial outcomes after the trial commenced, with reasons	7
Sample size	7a	How sample size was determined	7
	7b	When applicable, explanation of any interim analyses and stopping guidelines	NA
Randomisation:			
Sequence generation	8a	Method used to generate the random allocation sequence	7
	8b	Type of randomisation; details of any restriction (such as blocking and block size)	7
Allocation	9	Mechanism used to implement the random allocation sequence (such as sequentially numbered containers),	7

concealment mechanism		describing any steps taken to conceal the sequence until interventions were assigned	
Implementation	10	Who generated the random allocation sequence, who enrolled participants, and who assigned participants to interventions	7
Blinding	11a	If done, who was blinded after assignment to interventions (for example, participants, care providers, those assessing outcomes) and how	7
	11b	If relevant, description of the similarity of interventions	NA
Statistical methods	12a	Statistical methods used to compare groups for primary and secondary outcomes	8
	12b	Methods for additional analyses, such as subgroup analyses and adjusted analyses	8
**Results**			
Participant flow (a diagram is strongly recommended)	13a	For each group, the numbers of participants who were randomly assigned, received intended treatment, and were analysed for the primary outcome	8-9; 15-16
	13b	For each group, losses and exclusions after randomisation, together with reasons	8-9; 15-16
Recruitment	14a	Dates defining the periods of recruitment and follow-up	8
	14b	Why the trial ended or was stopped	NA
Baseline data	15	A table showing baseline demographic and clinical characteristics for each group	12-13
Numbers analysed	16	For each group, number of participants (denominator) included in each analysis and whether the analysis was by original assigned groups	9
Outcomes and estimation	17a	For each primary and secondary outcome, results for each group, and the estimated effect size and its precision (such as 95% confidence interval)	9-10
	17b	For binary outcomes, presentation of both absolute and relative effect sizes is recommended	9-10
Ancillary analyses	18	Results of any other analyses performed, including subgroup analyses and adjusted analyses, distinguishing pre-specified from exploratory	Supplementar y file
Harms	19	All important harms or unintended effects in each group (for specific guidance see CONSORT for harms)	NA
**Discussion**			
Limitations	20	Trial limitations, addressing sources of potential bias, imprecision, and, if relevant, multiplicity of analyses	10-11
Generalisability	21	Generalisability (external validity, applicability) of the trial findings	10-11
Interpretation	22	Interpretation consistent with results, balancing benefits and harms, and considering other relevant evidence	10-11
**Other information**			
Registration	23	Registration number and name of trial registry	3
Protocol	24	Where the full trial protocol can be accessed, if available	Supplementar

			y file
Funding	25	Sources of funding and other support (such as supply of drugs), role of funders	4

*We strongly recommend reading this statement in conjunction with the CONSORT 2010 Explanation and Elaboration for important clarifications on all the items. If relevant, we also recommend reading CONSORT extensions for cluster randomised trials, non-inferiority and equivalence trials, non-pharmacological treatments, herbal interventions, and pragmatic trials. Additional extensions are forthcoming: for those and for up to date references relevant to this checklist, see www.consort-statement.org.

Supplementary Data 3

Telephone Interview

1. **Could you tell me the name of the medical condition you have been diagnosed with and explain what it is in your own words?**

   ☐ Insufficient ☐ Sufficient ☐ Good ☐ Excellent

2. **Have you been prescribed new take-home medications in the Emergency Department for this medical condition?**

   ☐ Insufficient ☐ Sufficient ☐ Good ☐ Excellent

3a). (For those patients who have been prescribed new take-home medications) What are these drugs for and for how long should you take them? What are the possible complications of your medical condition?

☐ Insufficient ☐ Sufficient ☐ Good ☐ Excellent

3b). (For patients who have not been prescribed new take-home medications) Do you know why you have not been prescribed new medications? When are you going to have a follow-up visit? What are the possible complications of your medical condition?

☐ Insufficient ☐ Sufficient ☐ Good ☐ Excellent

4. **What symptoms would worry you enough to return to the Emergency Department?**
   ☐ Insufficient ☐ Sufficient ☐ Good ☐ Excellent

5. While in the Emergency Department did you get a chance to ask your doctor questions on your medical condition?

☐ No, none of them ☐ Only a few of them ☐ Nearly all I had in mind ☐ Yes, all of them

6. How would you assess the overall clarity of communication in the Emergency Department?

☐ Insufficient ☐ Sufficient ☐ Good ☐ Excellent

Patient's Level of Education (Years of Education): ☐ <5 ☐ 5-8 ☐ 8-13 ☐ >13

Supplementary Data 4

Interpretation of patients' answers

Question 1

Insufficient	Completely incorrect/Extremely vague information: the patient does not recall the technical term of the diagnosis **and** does not provide a logical description of it (both conditions are satisfied)	Examples: <<I do not know/ I do not remember>> - (AF) <<My heart doesn't beat well>>, (DVT) <<I have a swollen leg>>
Sufficient	The patient does not recall the technical term of the diagnosis; However, he/she provides a description of it, which contains only **some** logical elements.	Examples: (AF) <<The heart beats irregularly, perhaps because I am anxious>>, (DVT) <<I've got pain in my leg and it is swollen, maybe because I stay too long in bed>>
Good	The patient correctly recalls the technical term of his/her diagnosis, however he/she provides an incomplete/incorrect explanation of it - **or** –The patient provides an incomplete technical term of his/her medical condition, followed by a complete and logical description of it.	Examples: (AF) <<I have atrial fibrillation, which means the heart beats too fast>> or <<I've got fibrillation, which means the heart beats fast and irregular>> (DVT) <<I have a vein thrombosis, which means my veins are somehow damaged>> or <<I have a thrombosis, which means a clot obstructs blood flow in my arteries>>
Very good	Global/complete/correct description: the patient uses the technical term of the diagnosis and provides a logical description of it (both conditions must be satisfied).	Examples : (AF) <<I have atrial fibrillation, which means the heart beats at an irregular and fast pace>>, (DVT) <<I have a venous thrombosis, i.e. a clot obstructs blood flow in my vein>>

Question 2

Insufficient	The patient does not list any of the take-home medications prescribed<br><br>IF NO MEDICATIONS HAVE BEEN PRESCRIBED: The patient does not know at all whether or not new medications have been prescribed	
Sufficient	The patient recalls only some of the take-home medications prescribed and he/she provides incorrect dosage/does not recall the dosage.<br><br>IF NO MEDICATIONS HAVE BEEN PRESCRIBED: The patient provides an irresolute answer. He/she may mistake regular/chronic medications for new ones.	
Good	The patient lists all take-home medications but he/she does not remember their dosage; **<u>or</u>** The patient recalls the correct dosage of all medications but he/she cannot recall the name of all of them; **<u>or</u>** The patient correctly associates take-home medications with their dosage, however he/she does not remember all of them.<br><br>IF NO MEDICATIONS HAVE BEEN PRESCRIBED: The patient states that no new take-home medications have been prescribed, although he/she shows some hesitancy or insecurity.	
Very good	The patient lists all prescribed take-home medications and recalls the correct dosage for all of them.<br><br>IF NO MEDICATIONS HAVE BEEN PRESCRIBED: The patient knows for certain that no new take-home medications have been prescribed.	

Question 3a – (Please note: Take-home medications have been prescribed**)**

Insufficient	The patient does not know the purpose of the prescribed medications and does not know for how long he/she should take them.	
Sufficient	The patient knows either the purpose of the treatment **<u>or</u>** the duration of treatment **<u>or</u>** the technical term of the main complication of his/her condition (one of the three criteria must be satisfied)	**Please note**: the main complication is stroke for atrial fibrillation and pulmonary embolism for deep vein thrombosis.
Good	The patient knows two of the three criteria listed above (purpose of the medication/treatment duration/technical term of the main complication).	Examples (AF) <<I have to inject heparin to make my blood thinner until I see a cardiologist, otherwise I could get worse>><br><br>(DVT) <<I need to inject heparin to make my blood more fluid, in order to avoid a pulmonary embolism to

		occur; I can’t remember though for how long I should continue taking it>>.
Very good	The patient shows knowledge in all the criteria listed above (purpose of the medication/treatment duration/technical term of the main complication).	Example (AF) <<I have to take warfarin all my life to make blood thinner, otherwise clots might lead to a stroke>>

Question 3b – (Please note: No take-home medications have been prescribed**)**

Insufficient	The patient does not know why no therapy was prescribed; he/she does not know when the check-up will be; he/she does not know the technical term of the main complication of his/her medical condition (all three criteria must be met).	
Sufficient	The patient shows knowledge in at least one of the three above-mentioned criteria.	Example: (AF) <<I don’t know neither what complications I might have, nor why they haven’t prescribed anything at all; however, I’ll see a cardiologist next Monday and I guess he/she will explain that to me>>.
Good	The patient shows knowledge in at least two of the three above-mentioned criteria.	Example: (AF) <<I know I might develop a stroke, so I really can’t understand why they haven’t prescribed any blood thinners at all. Therefore, I have booked an appointment for tomorrow with my GP>>
Very good	The patient shows knowledge in all the three above-mentioned criteria.	Example (AF) << They didn't give me any blood thinners, because the fibrillation resolved and I am considered to be at low risk for recurrence; therefore, it’s unlikely I will develop a stroke. However, in a week I’ll see a cardiologist to keep the situation under control>>.

Question 4

Insufficient	The patient does not recall any symptom at all.
Sufficient	The patient names at least one of the symptoms listed below.
Good	The patient names at least 1/3 of the symptoms listed below.
Very good	The patient names at least 2/3 of the symptoms listed below.

**DVT**	**AF**
Dyspnoea/Shortness of breath	Palpitations/tachycardia
Chest pain	Bleeding (iatrogenic)
Sense of imminent death	Mental confusion
Tachycardia/palpitations	Syncope/Loss of consciousness
Syncope/Loss of consciousness	Visual disturbances/sudden drop in visus
Bleeding (iatrogenic)	Vertigo/dizziness/postural instability
	Speech disturbances
	Sensory disorders (altered sensation of a part of the body)
	Motor disorders (altered strength of a part of the body)

Question 5

☐ No, never ☐ A few of them ☐ Almost each one I had in mind ☐ Yes, all of them

Question 6

Please tell me how satisfied you think you are on a scale from zero to three.

Supplementary Data 5

Secondary Analyses

Per-protocol analysis for the primary outcome “Knowledge of the medical condition and its main complication”

```
Two-sample t test with equal variances
------------------------------------------------------------------------------
   Group |     Obs        Mean    Std. err.   Std. dev.   [95% conf. interval]
---------+--------------------------------------------------------------------
Standard |      69    6.130435    .4200399    3.489114    5.292258    6.968611
   Video |      68    8.779412    .4795399    3.954388    7.822246    9.736577
---------+--------------------------------------------------------------------
Combined |     137    7.445255    .3369858    3.944318    6.778846    8.111665
---------+--------------------------------------------------------------------
    diff |           -2.648977    .6369054                -3.90858   -1.389374
------------------------------------------------------------------------------
    diff = mean(Standard) - mean(Video)                           t =  -4.1591
H0: diff = 0                                     Degrees of freedom =      135

    Ha: diff < 0                 Ha: diff != 0                 Ha: diff > 0
 Pr(T < t) = 0.0000         Pr(|T| > |t|) = 0.0001          Pr(T > t) = 1.0000
```

Per-protocol analysis for the secondary outcome “Knowledge of the prescribed therapy”

```
Two-sample t test with equal variances
------------------------------------------------------------------------------
   Group |     Obs        Mean    Std. err.   Std. dev.   [95% conf. interval]
---------+--------------------------------------------------------------------
Standard |      69    3.115942    .2007406    1.667476     2.71537    3.516514
   Video |      68    3.397059    .2306038    1.901607    2.936772    3.857346
---------+--------------------------------------------------------------------
Combined |     137    3.255474    .1526316    1.786507    2.953636    3.557313
---------+--------------------------------------------------------------------
    diff |           -.2811168     .305443               -.8851891    .3229556
------------------------------------------------------------------------------
    diff = mean(Standard) - mean(Video)                           t =  -0.9204
H0: diff = 0                                     Degrees of freedom =      135

    Ha: diff < 0                 Ha: diff != 0                 Ha: diff > 0
 Pr(T < t) = 0.1795         Pr(|T| > |t|) = 0.3590          Pr(T > t) = 0.8205
```

Per-protocol analysis for the secondary outcome “Patient satisfaction”

```
Two-sample t test with equal variances
------------------------------------------------------------------------------
   Group |     Obs        Mean    Std. err.   Std. dev.   [95% conf. interval]
---------+--------------------------------------------------------------------
Standard |      69    7.666667    .4249601    3.529984    6.818672    8.514661
   Video |      68    8.441176    .3582603     2.95429    7.726086    9.156267
---------+--------------------------------------------------------------------
Combined |     137    8.051095     .279222     3.26821    7.498916    8.603273
---------+--------------------------------------------------------------------
    diff |           -.7745098    .5565456               -1.875186    .3261662
------------------------------------------------------------------------------
    diff = mean(Standard) - mean(Video)                           t =  -1.3916
H0: diff = 0                                     Degrees of freedom =      135

    Ha: diff < 0                 Ha: diff != 0                 Ha: diff > 0
 Pr(T < t) = 0.0832         Pr(|T| > |t|) = 0.1663          Pr(T > t) = 0.9168
```

Sensitivity analysis for the primary outcome "Knowledge of the medical condition and its main complication"

```
Two-sample t test with equal variances
------------------------------------------------------------------------------
   Group |     Obs        Mean    Std. err.   Std. dev.   [95% conf. interval]
---------+--------------------------------------------------------------------
Standard |      72    6.185219    .4036212    3.424839    5.380421    6.990017
   Video |      72    8.705292    .4541617     3.85369    7.799719    9.610865
---------+--------------------------------------------------------------------
Combined |     144    7.445255    .3205473    3.846567    6.811632    8.078879
---------+--------------------------------------------------------------------
    diff |           -2.520073     .607596               -3.721175   -1.318971
------------------------------------------------------------------------------
    diff = mean(Standard) - mean(Video)                           t =  -4.1476
H0: diff = 0                                     Degrees of freedom =      142

    Ha: diff < 0                 Ha: diff != 0                 Ha: diff > 0
 Pr(T < t) = 0.0000         Pr(|T| > |t|) = 0.0001          Pr(T > t) = 1.0000
```

Sensitivity analysis for the secondary outcome "Knowledge of the prescribed therapy"

```
Two-sample t test with equal variances
------------------------------------------------------------------------------
   Group |     Obs        Mean    Std. err.   Std. dev.   [95% conf. interval]
---------+--------------------------------------------------------------------
Standard |      72    3.121756    .1923459    1.632109    2.738229    3.505283
   Video |      72    3.389193    .2177362    1.847553    2.955039    3.823347
---------+--------------------------------------------------------------------
Combined |     144    3.255474     .145186    1.742232    2.968486    3.542463
---------+--------------------------------------------------------------------
    diff |           -.2674371    .2905271               -.8417544    .3068801
------------------------------------------------------------------------------
    diff = mean(Standard) - mean(Video)                           t =  -0.9205
H0: diff = 0                                     Degrees of freedom =      142

    Ha: diff < 0                 Ha: diff != 0                 Ha: diff > 0
 Pr(T < t) = 0.1794         Pr(|T| > |t|) = 0.3589          Pr(T > t) = 0.8206
```

Sensitivity analysis for the secondary outcome "Patient satisfaction"

```
Two-sample t test with equal variances
------------------------------------------------------------------------------
   Group |     Obs        Mean    Std. err.   Std. dev.   [95% conf. interval]
---------+--------------------------------------------------------------------
Standard |      72    7.682685    .4072308    3.455468    6.870689     8.49468
   Video |      72    8.419505     .338383    2.871275    7.744789    9.094222
---------+--------------------------------------------------------------------
Combined |     144    8.051095    .2656012    3.187215    7.526083    8.576107
---------+--------------------------------------------------------------------
    diff |           -.7368208    .5294714               -1.783486    .3098441
------------------------------------------------------------------------------
    diff = mean(Standard) - mean(Video)                           t =  -1.3916
H0: diff = 0                                     Degrees of freedom =      142

    Ha: diff < 0                 Ha: diff != 0                 Ha: diff > 0
 Pr(T < t) = 0.0831         Pr(|T| > |t|) = 0.1662          Pr(T > t) = 0.9169
```
